# Complete characterization of new isolates of *Neptunomonas phycophila* leads to emend its description and opens possibilities of biotechnological applications

**DOI:** 10.1002/mbo3.519

**Published:** 2017-09-18

**Authors:** Ana L. Diéguez, Phillip Pichon, Sabela Balboa, Thorolf Magnesen, Jesús L. Romalde

**Affiliations:** ^1^ Departamento de Microbiología y Parasitología CIBUS‐Facultad de Biología Universidade de Santiago Santiago de Compostela Spain; ^2^ Institute of Marine Sciences School of Biological Sciences University of Portsmouth Portsmouth UK; ^3^ Department of Biology Faculty of Mathematics and Natural Sciences University of Bergen Bergen Norway; ^4^ Present address: School of Biological Sciences University of Essex Wivenhoe Park Colchester UK

**Keywords:** aromatic compounds degradation, microbiota, *Neptunomonas phycophila*, *Pecten maximus*, WGS

## Abstract

Five strains were isolated from gonad of Great scallop (*Pecten maximus*) broodstock in a Norwegian hatchery. The study of 16S rRNA gene sequences showed that these isolates belong to *Neptunomonas phycophila*, a bacterium originally isolated from a symbiont of the anemone *Aiptasia tagetes* from Puerto Rico. The *gyr*B and *rpo*B genes sequences confirmed the affiliation of the scallop isolates to this species. Phenotypic characterization was performed and some differences between the Norwegian isolates and the type strain of *N. phycophila* were detected, such as ranges of temperature, pH, and tolerance to salinity or the use of several substrates as sole carbon source which lead to an emended description of the species. The strain 3CM2.5 showed phosphatidylethanolamine and phosphatidylglycerol as the major polar lipids. The whole genomes of the scallop strain 3CM2.5 and type strain of the species CECT 8716^T^ were obtained and the annotation of these genomes revealed the presence of genes involved in degradation of aromatic compounds in both strains. Results obtained not only widen the geographical and host ranges of *N. phycophila*, but also point out possible biotechnological applications for this bacterial species.

## 1 INTRODUCTION

The genus *Neptunomonas* was defined by Hedlund, Geiselbrecht, Bair, and Staley (1999) with the description of *Neptunomonas naphthovorans,* a bacterium implicated in degradation of polycyclic aromatic hydrocarbons (PAH) isolated from contaminated sediments. Bacteria of this genus belong to Family *Oceanospirillaceae* (Garrity, Bell, & Lilburn, 2005) within *Gammaproteobacteria*. Species of this group are Gram‐negative, facultatively aerobic, oxidase and catalase positive, and they can use several carbohydrates, sugar alcohols, organic acids, and some PAH as sole carbon and energy sources. In the last decade, six additional species have been described within this genus: *Neptunomonas japonica* (Miyazaki et al., 2008), *Neptunomonas antarctica* (Zhang et al., 2010), *Neptunomonas concharum* (Lee et al., 2012), *Neptunomonas qingdaonensis* (Liu et al., 2013), *Neptunomonas acidivorans* (Yang, Seo, Lee, Kim, & Kwon, 2014), and recently, *Neptunomonas phycophila* (Frommlet, Guimarães, Sousa, Serodio, & Alves, 2015), all of them isolated from marine environments. The latter species was described on the basis of a unique bacterial isolate associated to the dinoflagellate *Symbiodinium* species, a symbiont of the anemone *Aiptasia tagetes* in Puerto Rico.

During our studies on the microbiota of a marine hatchery of Great scallop (*Pecten maximus*) in Norway, five strains were isolated from broodstock gonad. These isolates, 3CM2.5, 3SH2.1, 3SM2.1, 2CH2.2, and 3CH2.4, were identified as *N. phycophila* by sequencing the 16S rRNA gene. In this study, phenotypic, genetic, and chemotaxonomic analyses were performed to fully characterize the Norwegian strains of *N. phycophila* which lead to an emended description of this species.

## 2 MATERIAL AND METHODS

### 2.1 Isolation and conservation of strains

All samples were obtained in January 2012 from broodstock gonad before and after spawning. Shellfish were opened aseptically by cutting the adductor muscle with a sterile scalpel. One gram of the gonad tissue was homogenized in 1 ml of artificial seawater (ASW). The mixture was serially diluted and 100 μl of each dilution were inoculated onto Marine Agar 2216 (MA; Difco). Plates were incubated at room temperature during 15 days. Different colonies were isolated as pure cultures and stored at −80°C in Marine Broth (MB; Pronadisa) supplemented with 20% (v/v) glycerol. Type strains of *Neptunomonas naphthovorans* CECT 7132^T^ and *N. phycophila* CECT 8716^T^ were obtained from the Colección Española de Cultivos Tipo (CECT) and included in the study for taxonomic comparison.

### 2.2 16S rRNA and housekeeping genes sequencing

Genomic DNA of pure cultures was obtained using the ‘Instagene’ matrix (Bio‐Rad), following the manufacturer's recommendations. Amplification of 16S rRNA gene of the different strains was carried out using primers 27F (5′‐AGAGTTTGATCCTGGCTCAG) and 1510R (5′‐GGTTACCTTGTTACGACTT) as previously described by Lane (1991). For the reference strains, sequences of 16S rRNA gene were retrieved from GenBank/EMBL. Sequence data analysis was performed with the DNASTAR Lasergene SEQMAN program. Sequences of the isolates were subjected to a BLAST search against EzTaxon‐extended database (Kim et al., 2012). Amplification of *gyr*B gene was performed using primers UP1 (5′‐GAAGTCATCATGACCGTTCTGC AYGCNGGNGGNAARTTYGA) and UP2r (5′‐AGCAGGGTACGGATGTGCGA GCCRTCNACRTCNGCRTCNGTCAT) according to Yamamoto and Harayama (1995) and amplification of *rpo*B gene was obtained with primers PasrpoB‐L (5′‐GCAGTGAAAGARTTCTTTGGTTC) and rpoB‐R (5′‐GTTGCATGTTNGNACCCAT) following the protocol described by Korczak, Christensen, Emler, Frey, and Kuhnert (2004). Sequences obtained for *gyr*B and *rpo*B genes were subjected to a BLAST search against the latest release of GenBank.

The phylogenetic trees based on 16S rRNA gene sequences and concatenated sequences of 16S rRNA and housekeeping genes were constructed with neighbor‐joining (NJ) and maximum‐likelihood (ML) algorithms, using the program MEGA version 6.0. (Tamura, Stecher, Peterson, Filipski, & Kumar, 2013). Distance matrices were calculated using Kimura's two‐parameter correction and stability of the groupings was estimated by bootstrap analysis (1000 pseudoreplicates).

### 2.3 Phenotypic characterization

The five scallop isolates together with the type strains of *N. phycophila* and *N. naphthovorans* were characterized by routine phenotypic tests following the methodologies described by Lemos, Toranzo, and Barja (1985); Romalde, Toranzo, and Barja (1990) and MacFaddin (1993). All media were supplemented with 1% NaCl when required. Catalase activity was determined based on bubble production in H_2_O_2_. The oxidase activity was determined by oxidation of 1% (w/v) *N*,*N*,*N*′,*N*′‐tetramethyl‐ρ‐phenylenediamine. Gelatinase and lipase activities and hydrolysis of aesculin and starch were analyzed on MA plates supplemented with 0.4%, 1%, 0.1%, and 0.4% (w/v) of substrates, respectively. Use of different compounds as a sole carbon source was tested on basal medium agar (BMA) supplemented with different substrates according to Baumann and Baumann (1981). A total of 48 enriched media, positive control (BMA and 5 g yeast extract) and negative control (BMA) were inoculated with 10 μl of each isolate suspension via a Denley Multipoint‐Inoculator (Denley Ltd) and incubated at 23°C for 14 days. Enriched media were examined daily using positive and negative control plates. Growth at various temperatures (4–44°C) was determined on MA plates. The optimum pH range for growth was examined in MB using appropriate biological buffers for adjusting the pH to 3, 5, 7, 9, 10, and 11. Tolerance to different saline concentrations was tested on basal medium (4 g/L neopeptone and 1 g/L yeast extract solidified with 1.5% (w/v) American bacteriological agar [Pronadisa]) supplemented with NaCl in a range from 0% to 10% and in sea salts solution (SIGMA) at concentrations between 0% and 15%, measuring cellular growth by turbidity (OD_600_). Additional biochemical tests for Norwegian strains were carried out using the miniaturized system API ZYM (bioMerieux) according to the manufacturer's instructions but using a medium containing artificial sea salts suspension (3.5% w/v). All these test and system were incubated at room temperature for 7 days.

### 2.4 Chemotaxonomy

Whole‐cell fatty acid analysis was concurrently performed on cells of five scallop isolates, *N. naphthovorans* CECT 7132^T^ and *N. phycophila* CECT 8716^T^ grown for 3 days at 25°C on MA (Difco). Extraction and analysis of whole‐cell fatty acids was carried out using the MIDI system in accordance with the protocols and instrument specifications recommended by the manufacturer (Sasser, 1990) using phospholipid fatty acids (PFLAD1) and environmental aerobes (TSBA) databases. Extraction and analysis of respiratory quinones and polar lipids of the strain 3CM2.5 were carried out by the identification service of the deutsche dammlung von mikroorganismen und zellkulturen (DSMZ) using thin‐layer chromatography following the methodology described by Tindall (1990a,1990bb).

### 2.5 Genome sequencing and annotation, DNA–DNA relatedness, average nucleotide identity and G+C content

In addition, DNA of isolates 3CM2.5 and *N. phycophila* CECT 8716^T^ were extracted with a High Pure PCR Template Preparation Kit (Roche) and sequenced at FISABIO Sequencing and Bioinformatics Service (Valencia, Spain) using Illumina MiSeq technology. Genome assembly was performed using SPAdes 3.6.2 (Nurk et al., 2013) and QUAST (Gurevich, Saveliev, Vyahhi, & Tesler, 2013). These whole‐genomes shotgun projects have been deposited at DDBJ/ENA/GenBank under the accession numbers of MRCJ00000000 (3CM2.5) and MRCI00000000 (*N. phycophila* CECT 8716^T^). The versions described in this paper are versions MRCJ01000000 and MRCI01000000, respectively.

The average nucleotide identity (ANI), using BLAST (ANIb) and MUMmer (ANIm) algorithms, was calculated using the software J‐species (V1.2.1) as described by Richter and Roselló‐Móra (2009). OrthoANI percentages were calculated as described by Lee, Kim, Park, and Chun (2015). *In silico* DNA‐DNA hybridization (DDH) values were estimated between strains 3CM2.5 and *N. phycophila* CECT 8716^T^ using the genome‐to‐genome calculator (GGDC2.0) (Auch, Klenk, & Göke, 2010bb; Auch, von Jan, Klenk, & Göker, 2010aa; Meier‐Kolthoff, Auch, Klenk, & Göker, 2013). The G+C mol% content of the strains was determined from the whole genome sequencing data. In addition, automatic gene annotation was carried out by the Rapid Annotations using Subsystems Technology (RAST) server (Aziz et al., 2008) and tRNAs were identified by tRNAscan‐SE v1.21 (Lowe & Eddy, 1997).

A sequenced‐based comparison analysis was performed using the RAST annotation server and Blast Ring Image Generator (BRIG; Alikhan, Petty, Ben Zakour, & Beatson, 2011) was used to obtain a genomic map showing similarity percentages. The upper and lower identity thresholds were set at 90% and 70%, respectively.

## 3 RESULTS

### 3.1 Polyphasic characterization of the isolates

**Figure 1:**
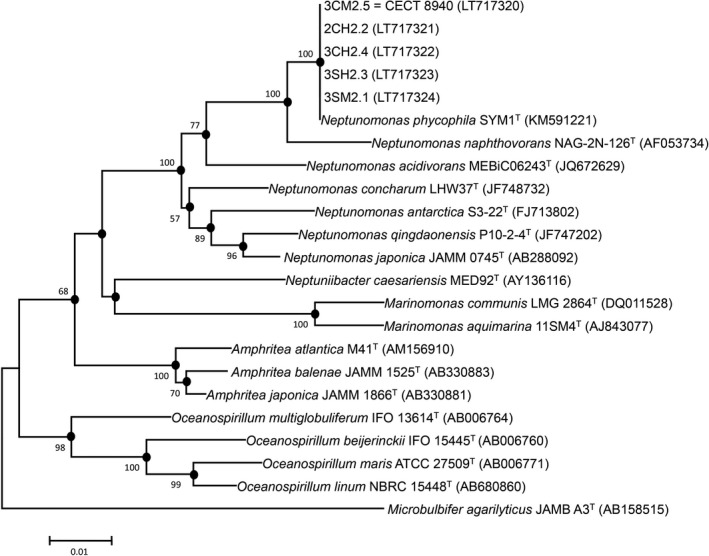
Neighbor‐joining tree based on 16S rRNA gene sequence data, showing the phylogenetic positions of strains within the genus *Neptunomonas*. Bootstrap values (expressed as percentages of 1,000 replications) greater than 50% are shown at the nodes. Black circles indicate nodes recovery in both neighbor‐joining and maximum‐likelihood trees. Bar represents 0.01 substitutions per nucleotide position

**Figure 2:**
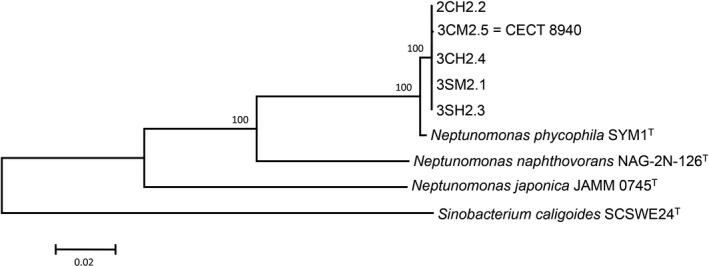
Neighbor‐joining tree based on concatenated sequences of 16S rRNA,* gyr*B, and *rpo*B genes. Bootstrap values (expressed as percentages of 1,000 replications) greater than 50% are shown at the nodes. Bar represents 0.02 substitutions per nucleotide position

According to the analysis of 16S rRNA gene sequences, the five isolates from great scallop showed a high similarity of 100% with the type strain of *N. phycophila* CECT 8716^T^. Phylogenetic tree based on these sequences showed that scallop isolates form a robust and homogeneous group together with *N. phycophila* within genus *Neptunomonas* (Figure 1). These results were complemented by the analysis of *gyr*B and *rpo*B genes as well as the concatenated sequences of these three genes which demonstrated the homogenicity of scallop isolates (Figure 2 and Figure S1). The Norwegian strains showed a similarity of 100% in their *gyr*B gene sequences, but this similarity dropped to 98.9% with the type strain of *N. phycophila*. Regarding to the similarity among the sequences of the *rpo*B gene, scallop isolates showed a similarity of 99.8%–100% among them, whereas the similarity with type strain ranged from 99.1% to 99.3%.

Phenotypically, Norwegian isolates exhibited convexed and glistening colonies with a white coloration and were approximately 0.8–1.5 mm in diameter and circular with opaque entire margins. These strains displayed homologous biochemical and morphological characteristics; being rod‐shaped, Gram‐negative, oxidase and catalase positive, and exhibiting oxidative carbohydrate metabolism. All isolates, with the exception of 3SM2.1 and 3CM2.5, were nonmotile, unlike *N. phycophila*‐type strain CECT 8716^T^ which is defined as a motile bacterium. Additionally, these isolates tested negative for gelatin, starch and aesculin hydrolysis, lipase and urease activity, indol and nitrate reduction. The range of growth at different temperature, NaCl concentration or pH observed for the scallop strains differed from those given for *N. phycophila* CECT 8716^T^. Temperature of growth for Norwegian isolates ranged between 4 and 37°C, with an optimum of 23°C. In addition, our isolates showed variable results of tolerance to saline concentrations. Unlike type strain of *N. phycophila*, scallop isolates were able to grow between 3% and 8% of NaCl except 2CH2.2 and 3CH2.4, which only grew to 6% of NaCl. Also, no isolate was able to grow with only 0.5% of NaCl. The pH growth range was 5–10 with the exception of 3SH2.3, which presented a pH range of 5–8, but contrarily to CECT 8716^T^ they don't grow at pH 11. Glucose was not fermented by scallop isolates, unlike *N. phycophila* CECT 8716^T^ which was able to weakly ferment several carbohydrates (Table 1).

**Table 1:** Characteristics of five scallop isolates and *Neptunomonas phycophila* CECT 8716^T^ a

	Scallop isolates	*N. phycophila* CECT 8716^T^
Morphology	Rod	Rod
Motility	v (2)^b^	+
Growth T^a^ (ºC)	4–37 (optimum 23)	4–40 (optimum 30)
Range pH	5.0–10	5.0–11
Range NaCl (%)	3–8	0.5–8
Fermentation of glucose	−	w^c^
*Use as Carbon source of*		
l‐Histidine	−	+
d‐Ribose	−	+
Arabinose	−	+
d‐Galactose	−	+
Trehalose	−	+
d‐Mannose	−	+
Cellobiose	−	+
Lactose	−	+
Gluconic Acid	+	‐
*trans*‐Aconitic Acid	v (1)	+
Tyrosine	v (1)	+
Putrescine	v (3)	−
Citruline	v (3)	−
*Enzymatic activity*		
Esterase (C4)	v (1)	−
Esterase lipase (C8)	v (2)	−
Lipase (C14)	v (1)	−
Valine arylamidase	v (4)	w
Cystine arylamidase	v (3)	−
Acid phosphatase	v (3)	+
Naphtol‐AS‐BI‐phosphohydrolase	v (1)	+

a All strains were positive for the use of d‐glucose, fructose, sucrose, d‐mannitol, *myo*‐inositol, glycerol, sodium acetate, pyruvate, l‐serine, d‐alanine, arginine, ornithine, propionic acid, citric acid, lactic acid, succinic acid, glutamic acid, amino‐*N*‐butyric acid, aspartic acid, β‐hydroxybutyric acid, fumaric acid, malic acid, and d‐saccharic acid and enzymatic activities of alkaline phosphatase and leucine arylamidase, whereas they were all negative for d‐xylose, l‐rhamnose, maltose, melibiose, salicin, amygdalin, sorbitol, *N*‐acetyl‐d‐glucosamine, glycine, l‐leucine, lysine, and threonine, and the enzymatics activities of trypsin, α‐chymotrypsin, α‐galactosidase, β‐galactosidase, α‐glucosidase, β‐glucosidase, β‐glucuronidase, *N*‐acetyl‐ β‐glucosaminidase, α‐mannosidase, and α‐fucosidase.

b v indicates variable results^.^ number of positive strains in brackets.

c w indicates a weak reaction.

Norwegian isolates showed extreme uniformity within the utilization of different compounds as sole carbon sources. Of the carbon sources tested, d‐ribose, arabinose, d‐xylose, d‐galactose, trehalose, d‐mannose, l‐rhamnose, maltose, cellobiose, lactose, melibiose, salicin, amygdalin, sorbitol, glycine, l‐leucine, threonine, l‐histidine, lysine, and *N*‐acetyl‐d‐glucosamine were not utilized by these isolates. Positive results were obtained for d‐glucose, fructose, sucrose, gluconic acid, d‐manitol, *myo*‐inositol, glycerol, sodium acetate, propionic acid, citric acid, pyruvate, lactic acid, succinic acid, l‐serine, glutamic acid, d‐alanine, arginine, ornithine, amino‐*N*‐butyric acid, aspartic acid, β‐hydroxybutyric acid, fumaric acid, malic acid, and d‐saccharic acid. Variable results were observed in the utilization of *trans*‐aconitic acid, tyrosine, citruline and putrescine. Type strain of *N. phycophila* CECT 8716^T^ showed different ability in the use of carbon sources being able to utilize d‐ribose, arabinose, d‐galactose, trehalose, d‐mannose, cellobiose, lactose, and l‐histidine but not gluconic acid (Table 1).

According to API ZYM, alkaline phosphatase and leucine arylamidase activity was present in all isolates, including type strain CECT 8716^T^, and trypsin, α‐chymotrypsin, α‐galactosidase, β‐galactosidase, β‐glucuronidase, α‐glucosidase, β‐glucosidase, *N*‐acetyl‐β‐glucosaminidase, α‐mannosidase, and α‐fucosidase activities were negative for all isolates. Variable results were detected in esterase (C4), esterase lipase (C8), Lipase (C14), valine arylamidase, cystine arylamidase, acid phosphatase, and naphtol‐AS‐BI‐phosphohydrolase (Table 1).

Major fatty acids for strain 3CM2.5 were Summed feature 8 (C_18:1_ω7c/C_18:1_ω6c), Summed feature 3 (C_16:1_ω7c/C_16:1_ω6c), C_16:0_, C_12:0_, and C_10:0_ 3OH. Similar values were obtained for strains 3SH2.3, 2CH2.2, 3CH2.4, 3SM2.1, and *N. phycophila* CECT 8716^T^ in this study. These results were slightly different to those given in *N. phycophila* description even though the conditions and growth medium were the same used by Frommlet et al. (2015) (Table 2). Summed Feature 3 is composed by C_16:1_ω7c/C_16:1_ω6c in our study, whereas in the description of *N. phycophila* contains C_16:1_ω7c/iso‐C_15:0_ 2‐OH. This last fatty acid was not detected in this study.

**Table 2:** Cellular fatty acid composition of the scallop strains and type strain of *Neptunomonas phycophila* CECT 8716^T^

Fatty acid	3CM2.5	3SH2.3	3SM2.1	3CH2.4	2CH2.2	CECT 8716^T^
C_10:0_ 3OH	5.07	4.25	5.24	4.55	4.06	5.83
C_12:0_	2.76	3.00	2.98	2.97	2.57	3.83
C_14:0_	*tr*	*tr*	ND	*tr*	*tr*	*tr*
C_16:0_	12.63	17.40	15.05	17.59	14.29	21.29
Summed feature 3	39.68	40.89	39.35	40.03	40.25	33.00
C_18:0_	*tr*	*tr*	*tr*	*tr*	*tr*	*tr*
Summed feature 8	37.65	32.81	36.68	33.86	37.10	33.52

Values are percentages of the total fatty acids; fatty acids that make up <1% of the total are indicated by *tr*. For unsaturated fatty acids, the position of the double bond is located by counting from the methyl (ω) end of the carbon chain. *cis*‐Isomer is indicated by the suffixes *c*. Summed features are groups of two fatty acids that cannot be separated by GLC with the MIDI system. Summed feature 3 contains C_16:1_ω7*c*/C_16:1_ω6*c* and Summed feature 8 contains C_18:1_ω7*c* and/or C_18:1_ω6*c*. All data obtained in the present study.

ND, not detected.

*In silico* DDH performed between 3CM2.5 and CECT 8716^T^ showed a result of 91.20% of similarity. Value of OrthoANI was of 98.98%. ANIb was 98.68%/98.93%, and ANIm was 98.99%, confirming that these isolates belonged to the same species.

The G+C content obtained for strain 3CM2.5 was of 45.3 mol% and 45.5 mol% for the strain CECT 8716^T^. This value is within the range defined for the different species of the genus *Neptunomonas,* which vary from 41.4 to 48.2 mol%.

The major polar lipids detected in isolate 3CM2.5 were phosphatidylethanolamine and phosphatidylglycerol. Also, unknown lipids, phospholipids, aminolipid, and glycolipid were detected (Figure S2). As for the type strain CECT 8716^T^, the principal respiratory quinone was Q8.

### 3.2 Emended description of *Neptunomonas phycophila* Frommlet 2015

The description of the *N. phycophila* is based on that given previously by Frommlet et al. (2015) but with the following changes. Bacteria form punctiform and adherent colonies with 0.5–1.5 mm in diameter on MA after 2 days of cultivation. Some strains of *N. phycophila* are not motile. Variable results for growth among strains at 40°C, pH 11 or 0.5% of NaCl were observed. Isolates can use d‐glucose, fructose, sucrose, d‐mannitol, *myo*‐inositol, glycerol, sodium acetate, pyruvate, l‐serine, d‐alanine, arginine, ornithine, propionic acid, citric acid, lactic acid, succinic acid, glutamic acid, amino‐*N*‐butyric acid, aspartic acid, β‐hydroxybutyric acid, fumaric acid, malic acid, and d‐saccharic acid but not d‐xylose, l‐rhamnose, maltose, melibiose, salicin, amygdalin, sorbitol, *N*‐acetyl‐d‐glucosamine, glycine, l‐leucine, lysine and threonine as sole carbon sources. Variable results were obtained for the use of D‐ribose, arabinose, D‐galactose, trehalose, D‐mannose, cellobiose, lactose, L‐histidine, gluconic acid, *trans*‐aconitic acid, tyrosine, putrescine and citruline.

Acid phosphatase, naphtol‐AS‐BI‐phosphohydrolase, esterase (C4), esterase lipase (C8), lipase (C14), valine arylamidase, and cystine arylamidase activities are variable. The G+C content of type strain of *N. phycophila* is 45.5 mol% and the range of G+C of the species is 45.3–45.5 mol%.

The major polar lipids of species are phosphatidylethanolamine and phosphatidylglycerol in addition to unknown lipid, phospholipid, aminolipid, and glycolipid.

### 3.3 Genomic analysis of *Neptunomonas phycophila*

**Figure 3:**
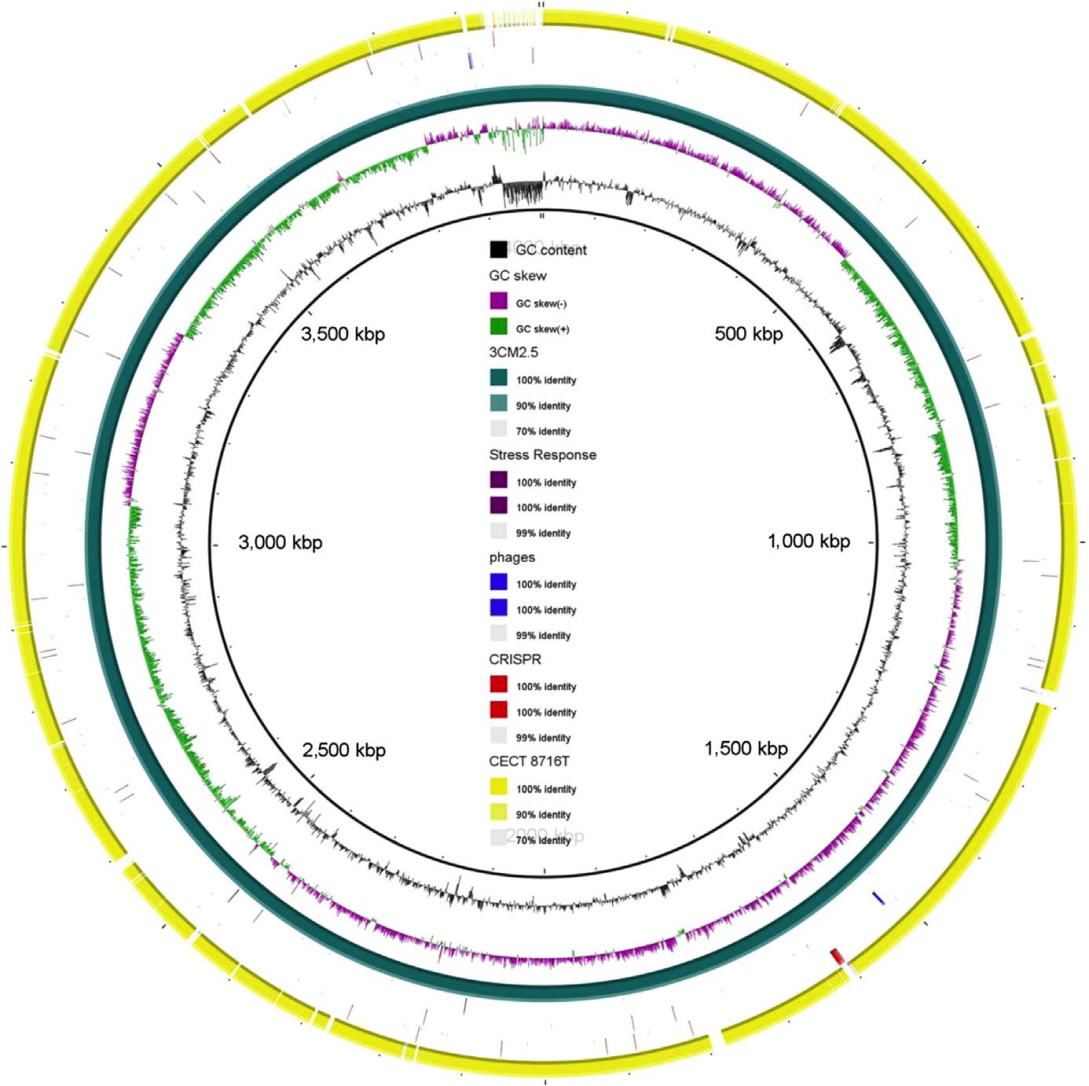
Graphical map of the BLASTN comparison of two strains of *Neptunomonas phycophila* genomes. From center to outside: GC content (ring 1), GC skew (ring 2), *N. phycophila* 3CM2.5 (ring 3), stress response genes (ring 4), phages genes (ring 5), CRISPR (ring 6), *N. phycophila* CECT 8716^T^ (ring 7).

Regarding whole genome sequence analysis, isolate 3CM2.5 showed a genome of 4,004,935 bp of size, with 287 contigs being the largest one of 519,039 bp in length, 3,646 coding sequences and 67 tRNAs. On the other hand, type strain CECT 8716^T^ showed a genome of 3,876,499 bp organized in only 27 contigs and 3,575 coding sequences. The largest contig had 3,052,931 bp in size and 60 tRNAs. Annotation of these genomes revealed the same subsystems in both isolates but with some important differences (Table 3; Figure 3). Thus, strain 3CM2.5 presented genes related with phage replication, phage capside proteins, and phage DNA synthesis which were not present in CECT 8716^T^. Also, strain 3CM2.5 showed 50 more subsystems of RNA metabolism than CECT 8716^T^. In addition, six CRISPRs were detected only in strain 3CM2.5. Another important difference among these genomes was in the presence of genes related to stress response. Isolate 3CM2.5 showed two genes associated to flavohemoglobin (fHb) and 40 sequences corresponding to bacterial hemoglobins which were absent in type strain CECT 8716^T^. The coded proteins are involved in responses to nitric oxide (NO) and nitrosative stress.

**Table 3:** Number and composition of subsystems in whole genome sequences of *Neptunomonas phycophila* strains CECT 8716^T^ and 3CM2.5

Subsystems	CECT 8716^T^	3CM2.5
Cofactors, vitamins, prosthetic groups, pigments	261	262
Cell wall, capsule	153	155
Virulence, disease, and defense	70	70
Potassium metabolism	19	19
Miscellaneous	44	44
Phages, prophages, transposable elements, plasmids	2	5
Membrane transport	166	157
Iron acquisition and metabolism	37	43
RNA metabolism	156	206
Nucleosides and nucleotides	87	88
Protein metabolism	263	266
Cell division and cell cycle	31	31
Motility and chemotaxis	105	105
Regulation and cell signaling	69	71
Secondary metabolism	6	6
DNA metabolism	101	98
Fatty acids, lipids, and isoprenoids	127	127
Nitrogen metabolism	36	36
Dormancy and sporulation	3	2
Respiration	107	106
Stress response	122	166
Metabolism of aromatic compounds	46	46
Amino acids and derivatives	416	418
Sulfur metabolism	17	18
Phosphorus metabolism	41	40
Carbohydrates	290	289

## 4 DISCUSSION

The genus *Neptunomonas* and its type species *N. naphthovorans* were described in 1999 to accommodate two strains isolated from creosote‐contaminated PAH degradation abilities (Hedlund et al., 1999). Between 2008 and 2014 five new species were added to the genus, all of them associated to marine environments or aquatic organisms, such as ark clams. The geographical range of these species not only includes Asiatic countries like China, Japan, or Korea, but also the Antarctica. During these years, the genus was subjected to two emended descriptions as new species were being described (Lee et al., 2012; Yang et al., 2014).

*Neptunomonas phycophila* was recently described on the basis of a unique bacterial strain isolated during a study of the microbiota associated with *in vitro* cultures of the dinoflagellate *Symbiodinium* sp. (Frommlet et al., 2015). To our knowledge, no further reference exists in the literature on this bacterial species. The isolates included in the present study, obtained from a Great scallop hatchery in Norway, were undoubtedly assigned to *N*. *phycophila* on the basis of their 16S rRNA gene sequence similarity, indicating that this bacterial species presents a wider host and geographical distribution, from the Caribbean to the North sea.

The scallop isolates and *N. phycophila* CECT 8716^T^ presented several differential characteristics as the temperature, pH, and salinity tolerance among others. The type strain of *N. phycophila* CECT 8716^T^ was described as able to grow at 40°C with an optimum of 30°C, whereas the five Norwegian isolates showed a lower range of temperature to growth, with an optimum of 23°C. One of the bacterial mechanisms of adaptation to changes in temperature is the modification of the fatty acid composition of their membranes. However, no differences were found between the two isolates in the analysis of the genes involved in cell membrane formation or in fatty acid biosynthesis. On the other hand, in this study, all isolates were grown under the same conditions of nutrients and temperature for the comparison of fatty acid composition. Thus, the differences in the profiles obtained were minimal, and could not be related to the variations in ranges of growth temperature among strains. The differences in the optimum growth temperatures exhibited by the strains might be due to an evolutionary adaptation of these bacteria to the distinct climate conditions of their habitat (Chattopadhyay, 2006) or, on the other hand, to the phenotypic plasticity of strains able to produce diverse phenotype characteristics in different environments (Sikorski, Brambilla, Kroppenstedt, & Tindall, 2008). Therefore, this variation should be in‐depth analyzed to determine if it is due to a transient change or an adaptation.

All the differences observed in the temperature, pH, salinity, utilization of carbon sources or enzymatic profiles made necessary an emended description of the species, which was originally performed on the basis of the analysis of a single strain.

As mentioned above, the type species of the genus *Neptunomonas, N. naphthovorans,* is related to the degradation of PAH. *N. phycophila* was not originally described as a degrading bacteria, and none of its representatives were obtained from contaminated waters, but our results demonstrated that genes involved in degradation of aromatic compounds are present in the genomes of its type strain CECT 8716^T^ as well as the scallop isolate 3CM2.5. Among others, genes implicated in catechol and chloroaromatic degradation pathways were detected in both genomes.

Another important difference between strains 3CM2.5 and CECT 8716^T^ was found in genes of the stress response in each of the strains. The 3CM2.5 isolate harbor two fHb and 40 hemoglobin sequences that are absent in the CECT 8716^T^ strain. These fHb are involved in the inactivation of NO by NO dioxygenase activity. NO is a molecule that modulates numerous aspects of animal physiology, including defense mechanisms in response to attack by microorganisms for which NO is toxic. However, in pathogenic bacteria the synthesis of fHbs that inactivate NO counteracts the defense mechanisms of infected cells.

In addition, sequences from bacteriophages and CRISPRs were detected only in strain 3CM2.5. Phages are the most abundant organisms in the biosphere and are widely represented in marine environments. The presence of these sequences in the 3CM2.5 strain might provide a kind of immune system against new infections (Koonin & Wolf, 2015). Also, the presence of several phage sequences can lead to increased genetic diversity and adaptation to different environments of bacterial populations (Rodríguez‐Valera et al., 2009), as well as conferring to the host certain advantages such as expression of resistance genes to antibiotics or even infective capacity (Davies, Winstanley, Fothergill, & James, 2016; Wagner & Waldor, 2002).

In conclusion, the results obtained in this study not only demonstrate an increased range of hosts and geographic distribution for *N. phycophila,* but also lead to update the description of this species. Our study also demonstrated that strains belonging to *N. phycophila*, as other representatives of the genus *Neptunomonas*, posses the genes necessary to degrade PAH compounds, opening an important field of research to properly characterize the degradative pathways in this bacterial species and to determine its potential biotechnological applications.

## CONFLICT OF INTEREST

All the authors declare that they have no conflicts of interest.
