# Supplementary material for: Complete characterization of new isolates of Neptunomonas phycophila leads to emend its description and opens possibilities of biotechnological applications
Source: Microbiologyopen. 2017 Sep 18;6(6):e00519. doi: 10.1002/mbo3.519 (PMC5727359; doi:10.1002/mbo3.519)

Complete characterization of new isolates of *Neptunomonas phycophila* leads to emend its description and opens possibilities of biotechnological applications.

A.L. Diéguez[1], P. Pichon[2], S. Balboa[1], T. Magnesen[3], J.L. Romalde[1]*.

[1]Departamento de Microbiología y Parasitología, CIBUS-Facultad de Biología, Universidade de Santiago, 15782 Santiago de Compostela, Spain.

[2] Institute of Marine Sciences. School of Biological Sciences. University of Portsmouth, PO4 9LY. Portsmouth, UK.

[3]Department of Biology. Faculty of Mathematics and Natural Sciences. University of Bergen. Bergen, Norway.

Supplementary Material

Figure S1: Neighbour-joining tree based on *gyr*B (a) and *rpo*B (b) genes sequences data, showing the phylogenetic positions of strains within the genus *Neptunomonas*. Bootstrap values (expressed as percentages of 1000 replications) greater than 50% are shown at the nodes. Bar, 0.05 substitutions per nucleotide position.

A

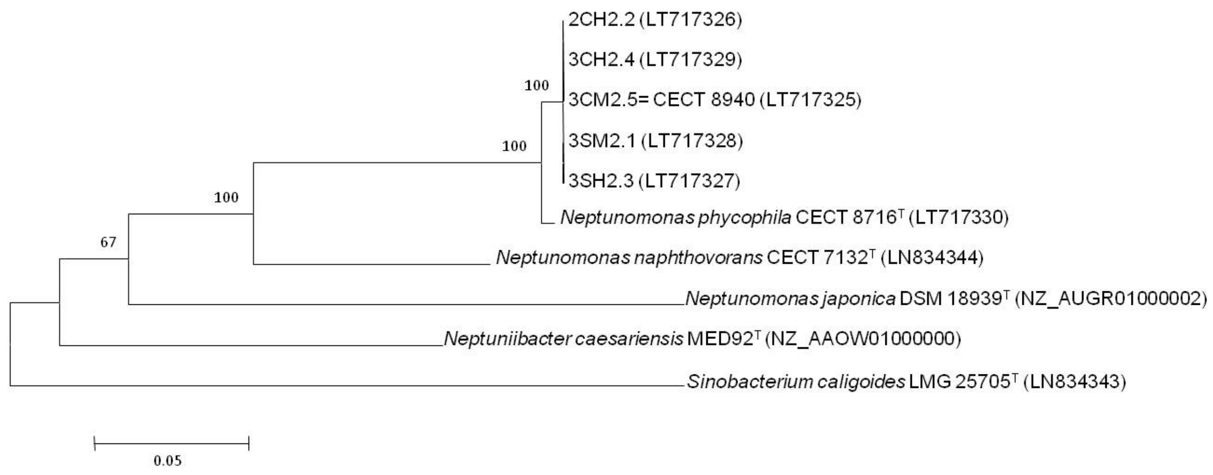

B

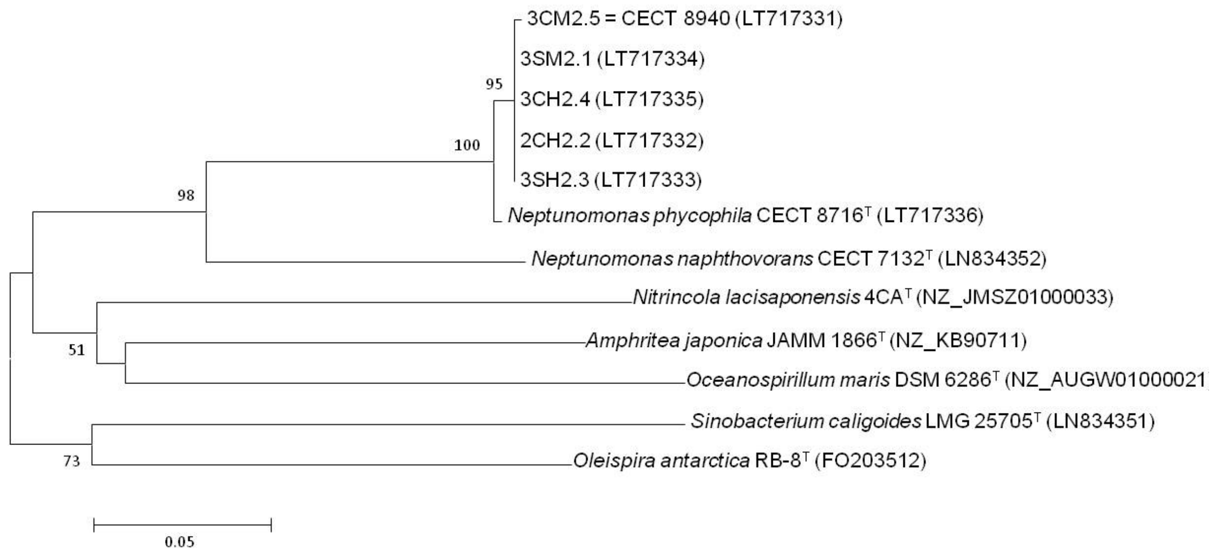

Figure S2. Polar lipid profiles of strain 3CM2.5. PE: Phosphatidylethanolamine; PG: Phosphatidylglycerol; PL: PhospholipidGL: Glycolipid; L: Lipid; AL: Aminolipid.

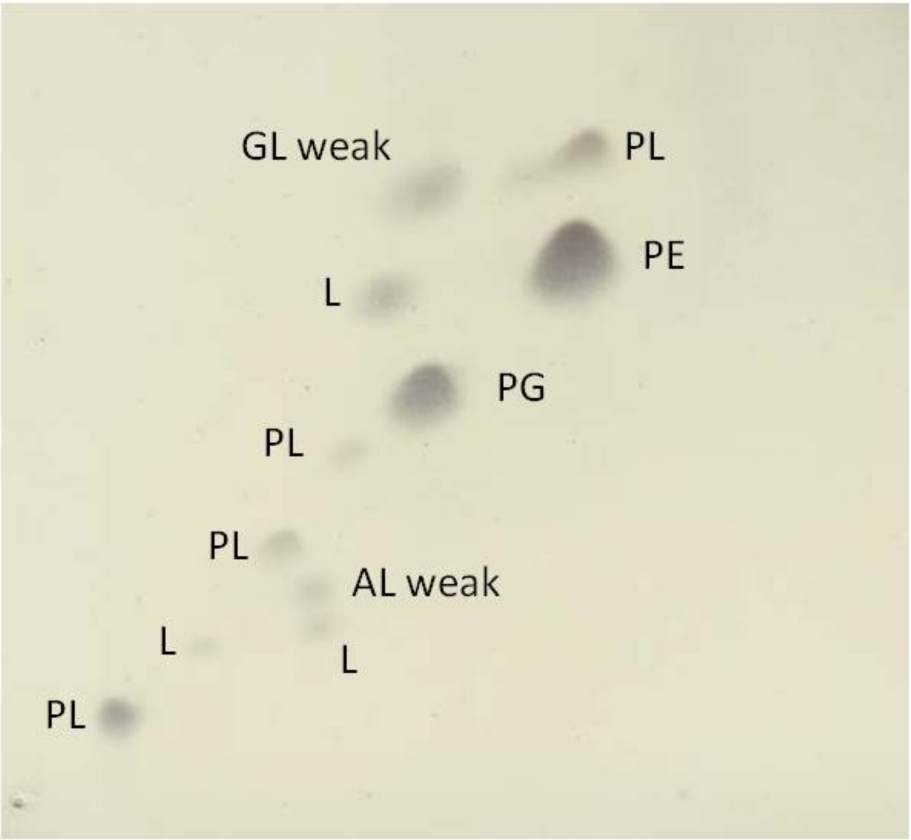

Supplement: Supplementary file 1 [file MBO3-6-na-s001.pdf]
